# Multiple vertebrae improves precision in image-based bone marrow absorbed dose estimation in [^177^Lu]Lu–DOTATATE treatment

**DOI:** 10.1186/s40658-026-00882-4

**Published:** 2026-05-09

**Authors:** Katja Smits, Frida Westerbergh, Jens Hemmingsson, Martijn van Essen, Johanna Svensson, Anna-Lena Theisen, Johannes Tran-Gia, Tobias Rydén, Peter Bernhardt

**Affiliations:** 1 Department of Medical Radiation Sciences, Institute of Clinical Sciences, Sahlgrenska Academy at University of Gothenburg, Gula Stråket 2B, 413 45 Gothenburg, Sweden; 2 Department of Clinical Physiology, Sahlgrenska University Hospital, Gothenburg, Sweden; 3 Department of Molecular and Clinical Medicine, Institute of Medicine, Sahlgrenska Academy at University of Gothenburg, Gothenburg, Sweden; 4 Department of Oncology, Institute of Clinical Sciences, Sahlgrenska Academy at University of Gothenburg, Gothenburg, Sweden; 5 Department of Nuclear Medicine, University Hospital Würzburg, Würzburg, Germany; 6 Medical Physics and Biomedical Engineering, Sahlgrenska University Hospital, Gothenburg, Sweden

**Keywords:** Bone marrow, Dosimetry, Lu-177, DOTATATE, SPECT

## Abstract

**Background:**

The bone marrow is a critical dose limiting organ in patients receiving radionuclide therapy, but image-based bone marrow dosimetry is not routinely performed in clinical practice. High noise levels and the limited number of vertebral cavities used in bone marrow absorbed dose calculations make absorbed dose estimates susceptible to large uncertainties. This study explores the effects of the number of included vertebrae, partial volume effects, and reconstruction parameters on image-based bone marrow absorbed dose calculations. We included sixteen patients with advanced neuroendocrine tumors treated with [^177^Lu]Lu–DOTATATE. Bone marrow absorbed dose estimates and their precision were analyzed based on the number of vertebrae included in the calculation. Additional evaluations were performed using both measured (Lung-Spine phantom with spherical inserts) and simulated (digital XCAT phantom) SPECT data, with noise levels matching patients’ bone marrow.

**Results:**

Increasing the number of vertebrae from one to six improved precision in absorbed doses, reducing the coefficient of variation (COV) from 34 to 6.2%. Only minor differences (0.1%) in bone marrow absorbed dose were observed when adjusting reconstruction parameters from 1 subset, 60 iterations, to 12 subsets, 5 iterations. In the Lung-Spine phantom, smaller volumes (4 mL) were more sensitive to noise than larger volumes (16 mL), with COVs of 36% and 17%, respectively, at 60 updates. In the XCAT phantom, a decrease in recovery was observed in vertebrae near high-uptake regions (L5: 0.63, L1: 0.29), at 60 updates at the highest noise level.

**Conclusion:**

Including multiple lesion-free vertebrae enhances precision in image-based bone marrow absorbed dose calculations. Nevertheless, careful selection of vertebrae is important, as closely located high-uptake areas can bias absorbed dose estimates. Applying partial volume correction could mitigate spill-out effects, further improving accuracy. Additionally, the minimal differences between reconstruction parameters suggest that bone marrow absorbed dose estimates remain stable across subset configurations.

**Supplementary Information:**

The online version contains supplementary material available at 10.1186/s40658-026-00882-4.

## Introduction

Patients with neuroendocrine tumors treated with a fixed posology of [^177^Lu]Lu–DOTATATE face a risk of hematologic toxicity; approximately 10% of all patients develop grade 3 or 4 toxicities, which can significantly limit further treatment options [1]. Despite the bone marrow being a critical absorbed dose (AD)-limiting organ, bone marrow dosimetry is not routinely conducted in clinical practice. Instead, blood sampling is commonly performed to monitor bone marrow function during therapy. Previous studies suggest that red marrow expresses somatostatin receptors, leading to specific uptake of [^177^Lu]Lu–DOTATATE, which is not accounted for in blood-based dosimetry [2, 3]. This implies that blood-based methods may underestimate the AD to the bone marrow. Image-based dosimetry could provide a more realistic and thus individualized assessment, offering a direct evaluation of bone marrow radiation burden and potentially enabling therapy adjustments. While significant correlations have been observed between image-based bone marrow AD and hematologic toxicity [4–7], these correlations remain too weak to reliably guide treatment decisions. Further methodological improvements are essential to establish image-based bone marrow dosimetry as a tool for personalized [^177^Lu]Lu–DOTATATE therapy, potentially improving treatment adaptation and long-term bone marrow health monitoring.

Image-based bone marrow dosimetry is often performed in the thoracic and lumbar vertebrae due to their large size and high red marrow content [8, 9]. However, image-based bone marrow dosimetry poses several challenges. The red marrow is heterogeneously spread in the trabecular bone cavities [10], with red marrow fractions varying between vertebrae depending on age, gender and physical status [9, 11]. In addition, the quantitative assessment of the activity concentration in the vertebrae can be interfered by spill-in [12], as well as by infiltrating skeletal metastasis.

One could argue that the biggest challenge in image-based bone marrow dosimetry is the low uptake and thus count rate found in the bone marrow, which is associated with a high noise level in the reconstructed SPECT. High noise levels in turn generate uncertainties in the quantification of the activity concentration, leading to uncertainties in the estimated bone marrow ADs. He and Frey investigated the effects of reduced acquisition times, i.e. increased noise, on the quantitative accuracy in numerous organs after peptide receptor radionuclide therapy [13] and concluded that the activity of larger organs is more likely to be accurately estimated and therefore less sensitive to noise. The reconstruction of noisy SPECT data plays a crucial role in the assessment of the bone marrow activity concentration. For SPECT projections with high noise levels, it has been suggested that reconstruction should be performed with fewer subsets to minimize noise in the SPECT images [14]. However, the influence of the number of subsets on noise levels and activity quantification in bone marrow dosimetry has not been systematically evaluated.

As SPECT imaging typically has a restricted field of view (FOV) and does not cover the entire skeleton, image-based dosimetry is often performed using a limited number of cavities as a surrogate for total bone marrow (summarized in Table 1). Furthermore, for time-saving reasons, SPECT data is often reconstructed using multiple subsets. However, it has not been fully explored how the number of segmented vertebrae and number of subsets in the reconstruction affect bone marrow AD estimation. The present study aimed to investigate the above mentioned aspects in patients with advanced neuroendocrine tumors treated with [^177^Lu]Lu–DOTATATE. For this purpose, the number of vertebrae evaluated (1–6) and the number of subsets used for reconstruction—while keeping the total number of updates constant (60i1s vs. 5i12s)—were varied, and the resulting bone marrow ADs were compared. In addition, a phantom analysis was conducted to assess how noise levels and the selection of vertebrae influence activity concentration estimates, using an Elliptical Lung-Spine phantom and a digital NURBS-based XCAT phantom.

**Table 1:** Summary of studies on/including image-based bone marrow dosimetry, detailing the total SPECT acquisition time, reconstruction parameters, included bone marrow sites, and whether RC correction was applied

Authors (Year)	SPECT acquisition time (min)	Reconstruction parameters	Type of reconstruction	Bone marrow sites	RC correction (Value)
Santoro (2018) [15]	22.5	6i10s	ACSCRR-OSEM	L2–L4	Yes (0.78)
Del Prete (2019) [16]	12–16	4i8s	ACSCRR-OSEM	L4–L5	No
Vergnaud (2022) [17]	15–40	8i8s	ACSCRR-OSEM	T9–L5	No
Hemmingsson (2023) [2]	20–30	6i10s	ACSCRR-OSEM	T9–L5, hip bone	No
Persson (2024) [7]	20	6i10s	ACSCRR-OSEM	L4	No
Blakkisrud (2024) [4]	25	48i1s	ACSCRR-O-S Conjugate gradient	Multiple in FOV	No
Present work	*20*–30	*60i1s 5i12s*	ACSCRR-OSEM	T8L5	No

*AC = Attenuation correction

*SC = Scatter correction

*RR = Resolution recovery

*OSEM = Ordered subset expectation maximization

## Materials & methods

### Patient study

This retrospective study was approved by the Swedish Ethics Review Board (registration number 2020-05092) and was performed in accordance with the Declaration of Helsinki and national regulations. The study included 16 patients, 10 women and 6 men, with advanced neuroendocrine tumors treated with [^177^Lu]Lu–DOTATATE according to the recommended scheme of 4 cycles of 7.4 GBq per cycle. The median patient age was 72 years (range 55–85 years). Patient data from the first treatment cycle was analyzed in this study. Six patients had confirmed bone marrow metastases based on pretherapeutic [^68^ Ga]Ga-DOTATATE PET/CT.

### Image acquisition and reconstruction

After the first therapeutic injection, four SPECT/CT images were acquired (2, 24, 48, and 168h post injection (h p.i.)). For two patients, the 48h p.i. scan was postponed to 120h p.i. One patient had the 168 h p.i. scan moved to 216 h p.i. All imaging was performed on two GE Discovery NM/CT 670 PRO SPECT/CT systems, equipped with 5/8″ NaI(TI) crystals and medium-energy general-purpose collimators. The photopeak window was centered at 208 keV ± 10%. SPECT projections were acquired using 120 projection views with 20–30s per projection. Reconstructions were performed using a Monte Carlo (MC) based ordered subset expectation maximization (OSEM) algorithm, that uses MC in the forward projection to compensate for scatter, attenuation, and the limited spatial resolution [18]. SPECT images were reconstructed using 60 total updates with two different iteration-subset combinations: 60 iterations with 1 subset (60i1s) and 5 iterations with 12 subsets (5i12s).

### Image analysis

**Fig. 1:**
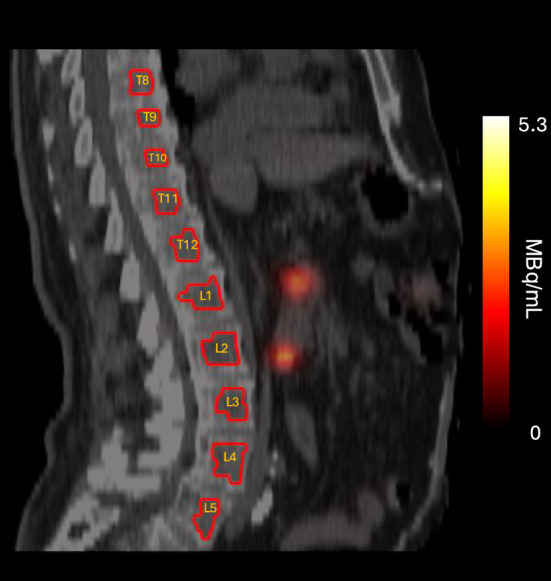
SPECT/CT image of patient K, with the thoracic and lumbar vertebrae VOI delineated in red.

Volumes-of-interest (VOIs) in the T8–L5 thoracic and lumbar vertebrae were manually delineated on the low-dose CT images for each time point p.i. (Fig. 1). Vertebrae with visible bone marrow metastases in T8–L5 were excluded (n = 5, 4% of all delineated vertebrae). Because of the typically limited FOV, vertebrae above T8 were not included. The activity concentration in the vertebral bone marrow was determined by extracting voxel values from each VOI and converting these values to activity concentration using camera and reconstruction-specific calibration factors*.* A large cylindrical VOI in a homogeneous cylindrical phantom was used to calculate the image calibration factor (ICF) as,

1 $$ ICF = \frac{{C_{cal} }}{{A_{cal} }} $$

where ${C}_{cal}$ is the count rate in the VOI and ${A}_{cal}$ is the activity in the phantom [19].

### Bone marrow dosimetry

Since there is no absolute reference for bone marrow ADs calculated in patients, our uncertainty analysis primarily evaluates precision rather than accuracy. In contrast, accuracy is assessed using phantom measurements, where a ground truth is available. The dependence of the precision in bone marrow AD on the number of vertebrae used in the AD calculations was evaluated as follows: For each patient, bone marrow ADs were calculated using the mean activity concentration in one to six vertebrae. For all available non-metastatic vertebrae, all possible unique combinations of smaller groups (from one to six vertebrae) were used for AD calculation. For example, when two vertebrae were used, the group included L4–L5, L3–L4, L2–L3 etc., (Suppl. Fig. 1). The number of available vertebrae in the patient cohort ranged from seven to ten, leading to different numbers of possible vertebrae combinations per patient. For instance, a total of 126 unique combinations were possible for seven vertebrae, while 847 combinations were possible for nine vertebrae. The number of unique vertebrae combinations per group ranged as follows: 7–9 for one vertebra, 21–45 for two, 35–120 for three, 35–210 for four, 21–253 for five, and 7–210 for six. To study the impact of adjacent high-uptake regions, bone marrows ADs were calculated using L3–L5 and compared with those calculated using T12–L2. Of the 16 included patients, 13 had no bone marrow metastases in T12–L5 and were therefore eligible for this analysis.

After biexponential time-activity curve fitting, the time-integrated activity was determined by integrating the function from zero to infinity. Since the human vertebra consists of trabecular bone along with both red and yellow marrow, a volume fraction of 0.574—representing the proportion of the vertebra occupied by active red marrow in lumbar and thoracic regions (average for men and women)—was used for AD calculation [20]. In addition, an absorbed fraction of 0.645, representing the proportion of energy emitted within the red marrow that is absorbed by the red marrow itself, was applied [20]. The density of bone marrow was set to 1.03 × 10^−3^ kg/mL [21]. The AD from ^177^Lu photon emissions was neglected. AD calculations were performed using MATLAB version 9.13.0 (R2022b) (The MathWorks Inc, Natick, Massachusetts, United States of America).

To study how bone marrow AD depends on the number of subsets in the reconstruction, differences in bone marrow AD (∆AD) were calculated based on the number of subsets as,

2 $$ \Delta AD = \frac{{AD_{5i12s} - AD_{60i1s} }}{{AD_{5i12s} }} $$

The precision of bone marrow AD for each patient was measured using the coefficient of variation (COV). The COVs were determined as

3 $$ {\mathrm{COV}} = \frac{\sigma }{\mu } \cdot 100 $$

where σ is the standard deviation of the group of unique vertebrae combinations (for example, the group with one vertebra used in the AD calculations), and μ is the mean value of the same group.

### Phantom studies

To systematically assess the impact of the number of updates (iterations x subsets) on bone marrow activity quantification, all phantoms were reconstructed with the same OSEM algorithm used for patient imaging, with update numbers ranging from 12 to 204, while keeping the subset number fixed at 12 (1i12s–17i12s).

### Anthropomorphic elliptical lung-spine phantom

To examine the impact of noise on bone marrow activity quantification, an Elliptical Lung-Spine Phantom™ was used. The phantom contains six spherical inserts (0.5–16 mL), two lung inserts with styrofoam beads, and a spinal rod insert. The phantom was filled with a sphere-to-background ratio of 2:1 (activity concentration 0.58 MBq/mL of ^177^Lu in the spheres) to mimic the bone marrow-to-background ratio observed in patient SPECT images acquired 2h post injection. Diethylenetriaminepentaacetic acid (DTPA) was added to stock solutions to prevent ^177^Lu from sticking to the phantom walls. The phantom was imaged on one of the SPECT/CT cameras used in the patient study, with 120 projection views at 600s per projection (total acquisition time: 10h), and list mode data was extracted.

### Generating noise levels

Realistic noise levels were determined by calculating the activity concentration in each vertebra 24h p.i. from patients without bone metastases. Three representative activity concentrations were identified: the maximum, median, and minimum values among patients. Corresponding noise levels (η_min_, η_med_, and η_max_, respectively) were then generated to match these activity concentrations. This approach ensured that the simulated noise levels were reflective of the range of activity concentrations found in patient vertebrae, providing a realistic basis for further analysis. For each noise level, 100 noise realizations (projection datasets of matrix size 128 × 128x120) were produced from the list-mode file (Suppl. Table 1). To examine the impact of reducing the number of subsets on noisy images, the highest noise level (η_max_) was reconstructed with update numbers ranging from 12 to 204, while keeping the subset number fixed at 1 (12i1s–204i1s). This approach was designed as a counterpoint to the reconstruction protocol employing 12 subsets and varying update numbers from 1 to 17 (1i12s–17i12s) described above.

### Analysis

The three largest spheres (4–16 mL) in the phantom were included in the analysis, as they matched the general size of patients’ vertebral VOIs. Spherical VOIs, corresponding to the size of each insert, were defined, and the activity concentration in each VOI for each noise realization was calculated using the same approach as for the patient vertebra. The recovery coefficients (*RC*) were calculated for all noise realizations, as

4 $$ RC\left( v \right) = \frac{{C_{R} \left( v \right)}}{{ICF \cdot A_{R} \left( v \right)}} $$

where ${C}_{R}\left(v\right)$ is the measured count rate for each sphere $v$, and ${A}_{R}\left(v\right)$ is the true sphere activity in the insert. For each update, the precision of the RCs was calculated using the COV, defined as the standard deviation of all noise realizations divided by their mean value (Eq. 3).

## Digital phantom

### XCAT Phantom

In addition to the physical phantom, the digital NURBS-based XCAT phantom was used to evaluate how bone marrow activity concentration estimations vary with a more complex source geometry. The uptake in kidneys, spleen, liver, lungs, and bone marrow were determined for all patients. The background activity was estimated by measuring the activity concentration in subcutaneous adipose tissue located near the hip bone. One of the 16 patients without bone metastases, whose estimated activity distribution closely matched the cohort median for all relevant organs, was chosen to represent the activity distribution in the XCAT phantom. Four activity maps, each corresponding to one of the patient’s four imaging time-points, were generated. All regions, including the entire bone marrow, were modelled using a homogeneous activity distribution (Suppl. Table 2). Additionally, to study the impact of spill-in from high-uptake regions close to the vertebrae, a separate activity map was created with only activity in the bone marrow (5 in bone marrow, 1 in background).

Using the aforementioned in-house MC-based code, noise-free ^177^Lu SPECT projections of the 208 keV photopeak window (187.2–228.8 keV) were simulated (120 projections, 128 × 128 matrix size), using 21 million photon histories [18]. The camera parameters used for modelling were chosen to replicate the SPECT/CT system used for the patients included in the study. Simulations were performed using an energy resolution of 9.5% with an intrinsic spatial resolution of 4.5 mm.

### Generating noise levels

To mimic the noise levels observed in patients, Poisson-distributed noise was added to the noise-free projections. This was done by normalizing the count level to the mean found in the patient’s right kidney for each corresponding time point, and then randomly rearranging the voxel values according to a Poisson distribution. One hundred noise realizations were generated for each time point p.i.

### Analysis

VOIs were manually delineated in the XCAT vertebral bone marrow from T12 to L5. The voxel values in the XCAT vertebrae of the noisy SPECT reconstruction were compared to those of the activity map, by computing recovery as the ratio of the noisy SPECT reconstruction to the true activity values. The precision in recovery for each update was calculated using the COV as above. Recovery and COV were calculated for each time point p.i., comparing the use of only L5 with the use of multiple vertebrae (L4–L5, L3–L5, L2–L5, L1–L5, and T12–L5).

In addition, because the ground truth is available, the accuracy in bone marrow AD was evaluated using the XCAT phantom. The bone marrow AD in the XCAT phantom was calculated for combinations L3–L5 and L2–T12, by taking the mean recovery for these vertebrae combinations for each time point and deriving the mean activity concentration. The bone marrow AD was calculated in the same manner as the patients described above. The calculated bone marrow AD for L3–L5 and T12–L2 were then compared to the ground truth bone marrow AD, i.e. when the recovery in the vertebrae is equal to one, for vertebrae combinations L3–L5 and L2–T12, respectively.

### Statistical analysis

The statistical analysis was preformed using Microsoft Excel Version 16.88 (Microsoft Corporation, Redmond, Washington, United States). To compare bone marrow ADs calculated using different numbers of subsets, the mean AD for all patients across the six groups, each defined by the number of vertebrae used for AD calculations, was compared between images reconstructed with 1 subset (60i1s) and those reconstructed with 12 subsets (5i12s). Two-sample t-tests were performed, with a Bonferroni correction, between the different groups (*p* < 0.01). A paired t-test (p = 0.05) was performed to compare activity concentration as well as bone marrow AD calculated using L3–L5 versus T12–L2. The mean recovery of the XCAT phantom vertebrae in L3–L5 was compared with T12–L2 for all time points with a two-sample t-test.

## Results

### Patient study

**Fig. 2:**
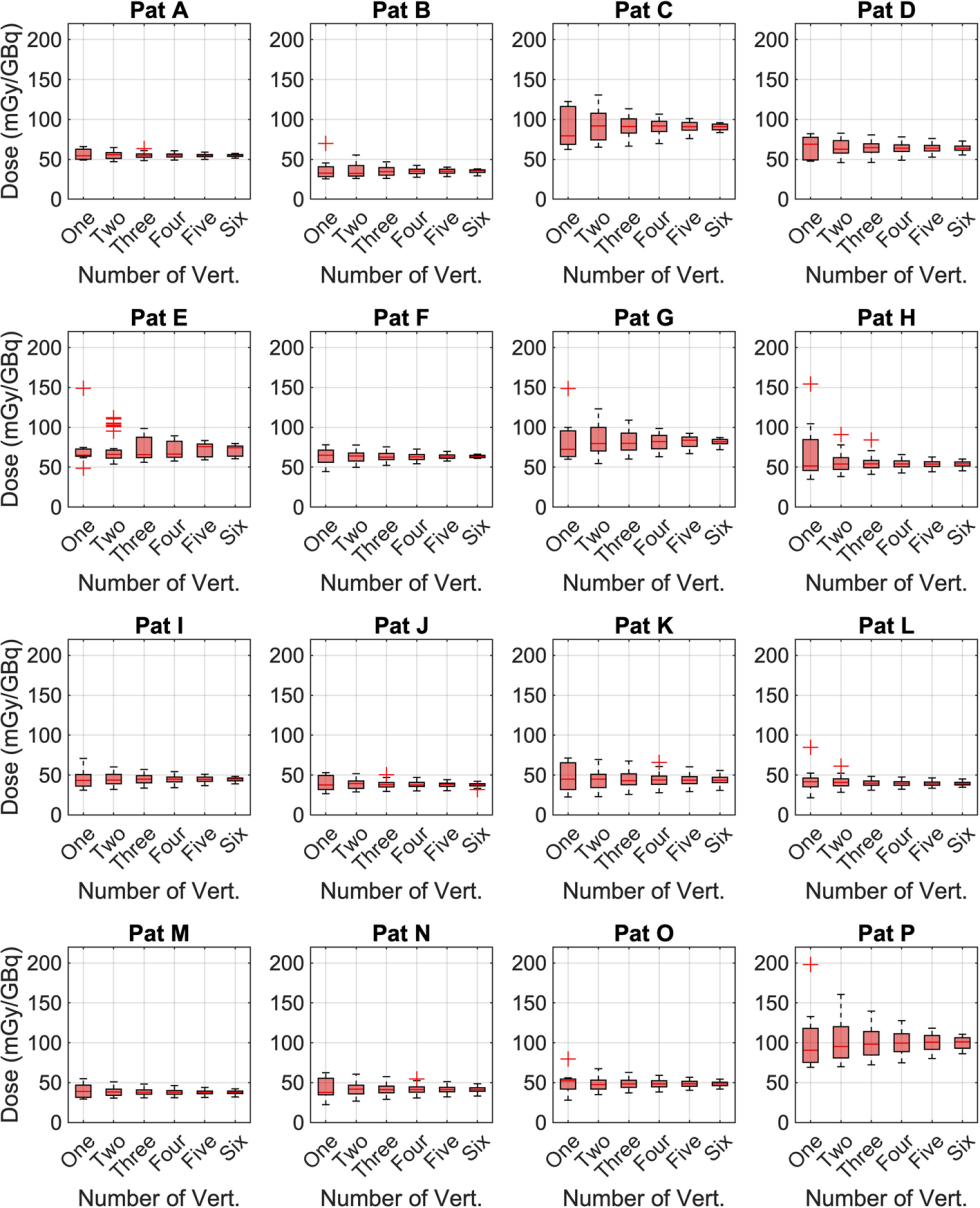
Bone marrow absorbed doses for all patients, calculated using one to six vertebrae with 60 updates and 1 subset (60i1s), are presented. Variations in bone marrow absorbed dose, serving as an indicator of precision, are displayed as boxplots. Outliers are represented by individual points beyond the whiskers.

Generally, across the 16 patients, the precision of mean bone marrow AD improved as more vertebrae were included in the calculations (Fig. 2). The COV (as a measure of precision) in bone marrow AD, calculated using one to six vertebrae, progressively improved from 32 to 19%, 14%, 10%, 8.2%, and finally 6.2% across all patients (Table 2). A decrease in mean bone marrow AD across all patients was observed when comparing values calculated using L3–L5 (50.93 mGy/GBq) versus T12–L2 (47.49 mGy/GBq). However, the differences in bone marrow AD and activity concentrations between L3–L5 and T12–L2 were not statistically significant. Similarly, no significant difference was found when comparing the ADs reconstructed with 1 and 12 subsets, for all patients (Suppl. Table 3). The mean difference in AD reconstructed with 1 and 12 subsets was −0.1% (−3.5–5.3%) (Suppl. Table 4).

**Table 2:** Mean bone marrow absorbed doses for each patient, calculated using one to six vertebrae with 60 updates, 1 subset (60i1s), are presented in mGy/GBq. The coefficient of variation (COV), as a measure of precision, is provided in brackets. The variable *n* denotes the number of vertebrae delineated on each patient.

	Dose (COV) [mGy/GBq]					
	One vert	Two vert	Three vert	Four vert	Five vert	Six vert
Pat A (*n* = 8)	56.1 (12%)	55.2 (8.3%)	54.8 (6.1%)	54.8 (4.8%)	54.7 (3.8%)	54.6 (2.7%)
Pat B (*n* = 8)	37.3 (39%)	35.4 (23%)	35.1 (16%)	34.9 (12%)	34.8 (9.1%)	35.0 (6.7%)
Pat C (*n* = 7)	89.7 (29%)	92.4 (20%)	91.3 (14%)	91.1 (10%)	90.8 (7.3%)	90.5 (4.9%)
Pat D (*n* = 9)	64.5 (22%)	64.4 (15%)	64.2 (12%)	64.0 (9.1%)	63.9 (7.4%)	63.9 (5.9%)
Pat E (*n* = 9)	74.3 (39%)	72.5 (25%)	72.0 (18%)	71.9 (14%)	71.6 (11%)	71.5 (9.0%)
Pat F (*n* = 7)	63.2 (18%)	63.3 (12%)	63.1 (9.0%)	63.1 (6.9%)	63.2 (4.8%)	63.4 (3.0%)
Pat G (*n* = 7)	85.0 (37%)	83.7 (22%)	81.7 (17%)	81.8 (12%)	81.8 (9.1%)	81.4 (6.1%)
Pat H (*n* = 9)	69.9 (54%)	55.0 (20%)	54.4 (14%)	54.0 (10%)	54.0 (7.9%)	53.9 (6.3%)
Pat I (*n* = 8)	45.3 (28%)	44.7 (17%)	44.6 (13%)	44.7 (10%)	44.6 (7.7%)	44.7 (5.5%)
Pat J (*n* = 9)	39.0 (24%)	38.4 (16%)	38.0 (12%)	38.0 (10%)	37.9 (7.7%)	37.8 (6.2%)
Pat K (*n* = 10)	46.8 (41%)	44.2 (27%)	43.9 (21%)	43.8 (17%)	43.7 (14%)	43.7 (11%)
Pat L (*n* = 10)	43.6 (39%)	41.0 (16%)	39.7 (10%)	39.4 (8.4%)	39.3 (6.9%)	39.2 (5.6%)
Pat M (n*n*= 10)	39.7 (22%)	38.4 (13%)	38.0 (10%)	37.9 (8.3%)	37.8 (6.7%)	37.8 (5.5%)
Pat N (*n* = 10)	42.3 (31%)	41.4 (19%)	41.5 (14%)	41.4 (11%)	41.4 (9.3%)	41.4 (7.6%)
Pat O (*n* = 9)	49.9 (29%)	48.6 (18%)	48.7 (13%)	48.5 (10%)	48.5 (8.1%)	48.4 (6.3%)
Pat P (*n* = 8)	104.4 (41%)	101 (25%)	100 (18%)	100 (14%)	100 (10%)	100 (7.4%)
Mean	59.4 (32%)	57.5 (19%)	57.0 (14%)	56.8 (10%)	56.8 (8.2%)	56.7 (6.2%)

### Measured SPECT data (lung-spine phantom)

**Fig. 3:**
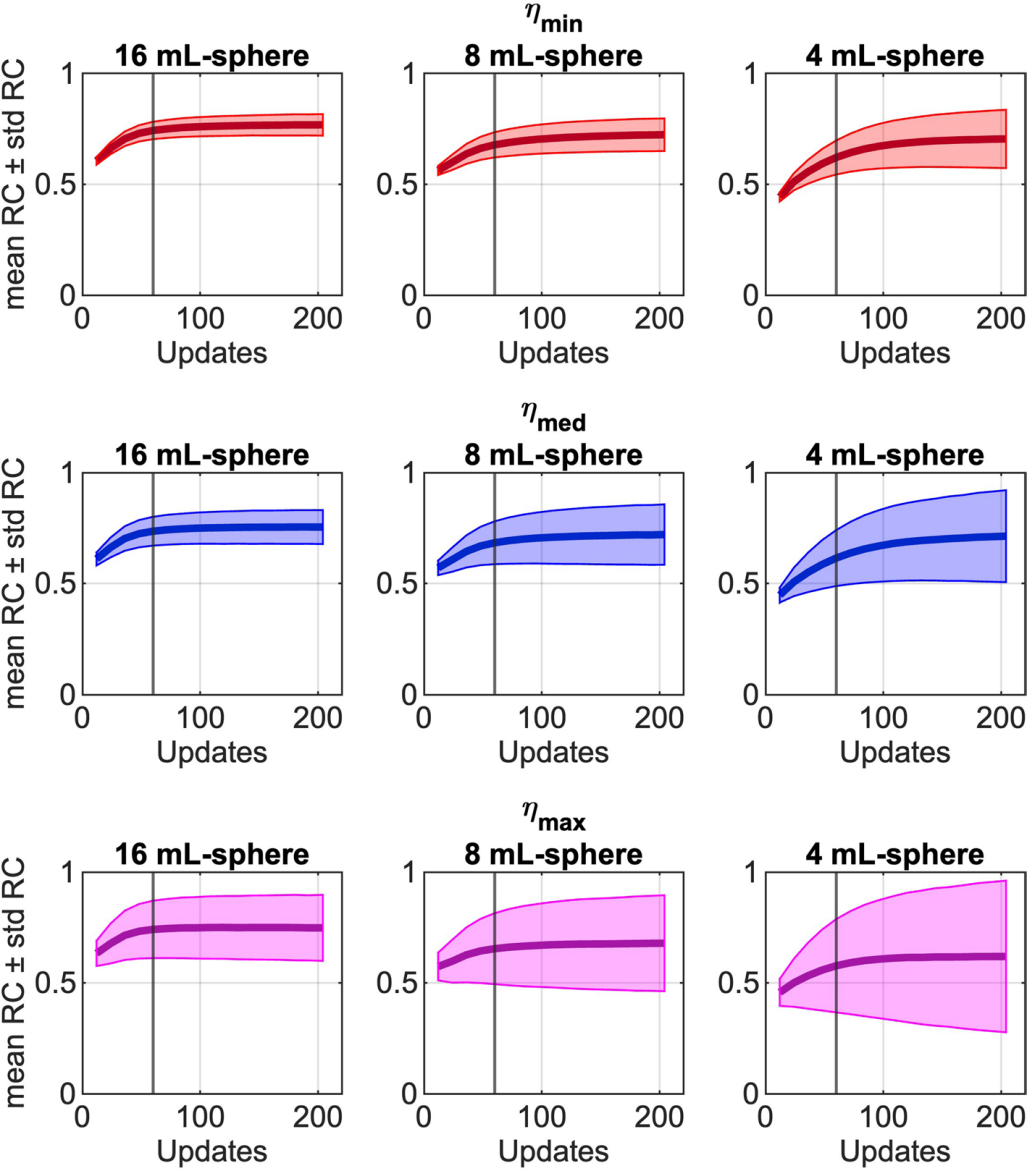
Mean recovery coefficient (thick line) and standard deviation (thin line) of the recovery coefficient of the noise levels η_min_ (red), η_med_ (blue), and η_max_ (magenta), for the three different sphere sizes (4, 8, and 16 mL), in the Lung-Spine phantom. The noise realizations are all reconstructed using 12–204 updates, with 12 subsets (1i12s–17i12s, respectively). Black vertical line shows 60 updates.

Figure 3 shows the precision of RCs across increasing noise levels (top to bottom) for the Lung-Spine phantom. At 60 updates (black vertical line)—the same number of updates as in the patient study—the precision of RC for the three spheres decreased, with variability increasing from 5–12 to 17–36% as noise levels increased (Suppl. Table 5). Similarly, precision decreased for smaller spheres and when increasing the number of updates. The mean RC (0.74) remained stable across all noise levels for the 16 mL sphere (Suppl. Table 5). However, for the 8 mL and 4 mL spheres, RC decreased from 0.68 and 0.62 to 0.65 and 0.58, respectively, as noise increased.

At the highest observed noise level in the Lung-Spine phantom (η_max_), small differences in mean RC and precision were observed when comparing images reconstructed with 1 subset (204i1s) and 12 subsets (17i12s) (Suppl. Fig. 2 and Table 6).

### Simulated SPECT data (XCAT phantom)

**Fig. 4:**
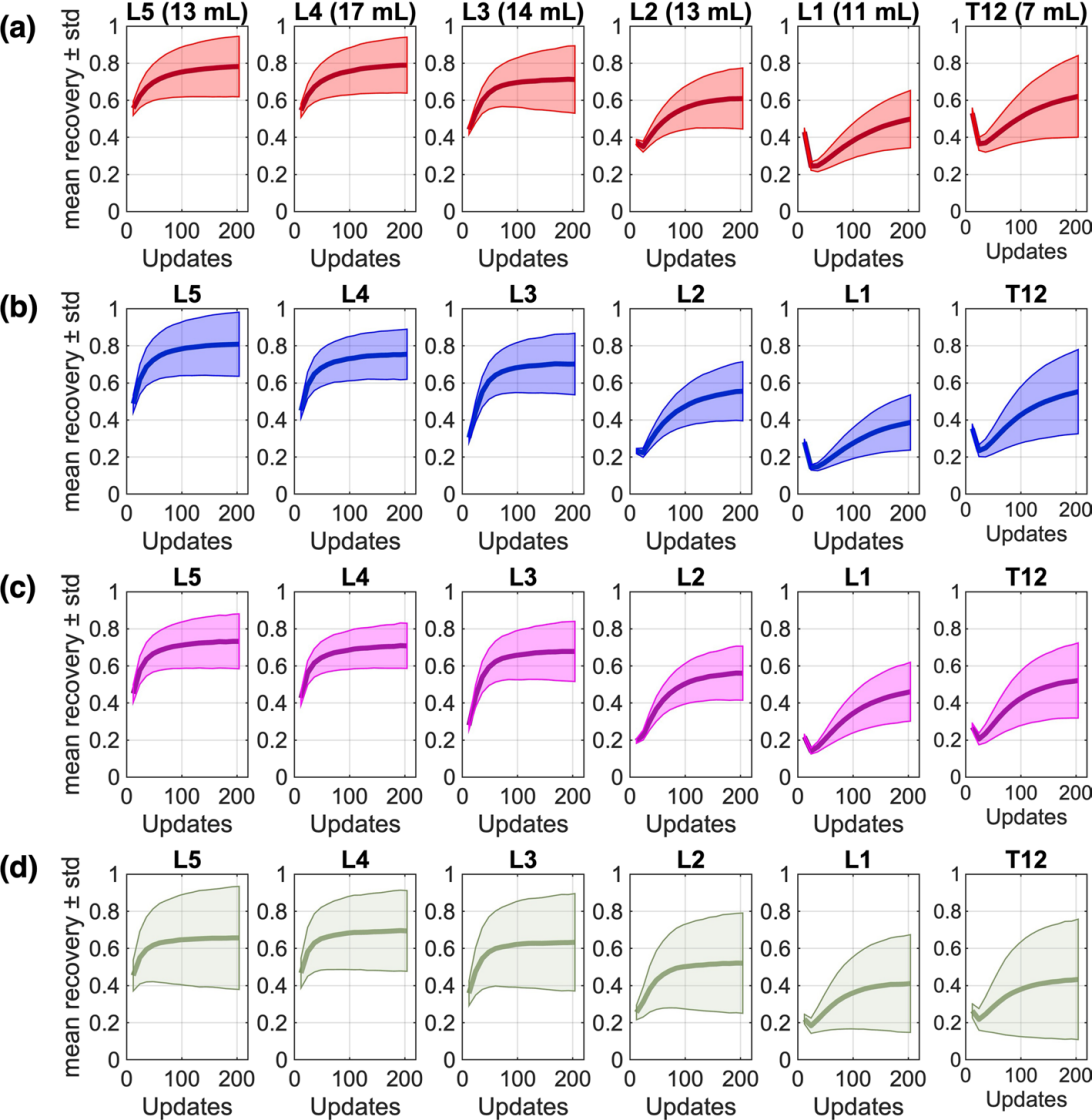
Mean recovery (thick line) and standard deviation (thin line) of the recovery of XCAT vertebrae voxel values across different noise realizations, for each individual vertebra. Four XCAT activity maps **a** 2h p.i., **b** 24h p.i., **c** 48h p.i., and **d** 168h p.i. are presented. All noise realizations were reconstructed using 12–204 updates, with 12 subsets (1i12s–17i12s, respectively).

**Fig. 5:**
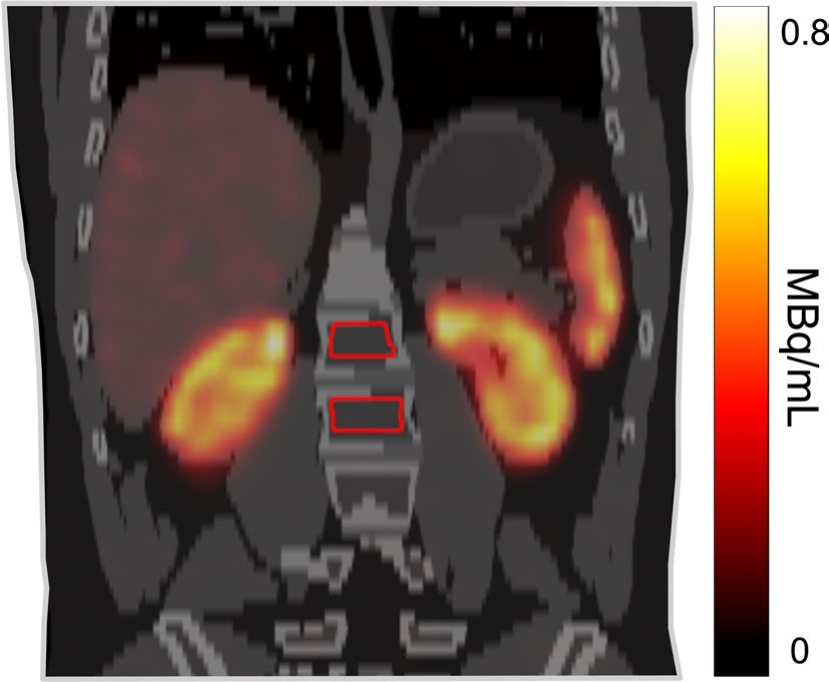
The XCAT activity (color scale) and attenuation map (gray scale) at 24h p.i., with vertebrae L1 and L2 delineated in red.

**Fig. 6:**
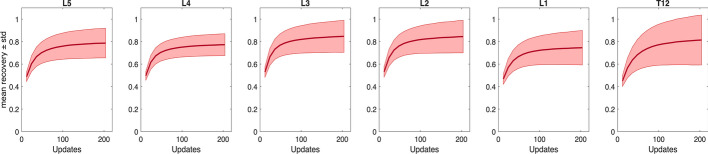
Mean recovery (thick line) and standard deviation (thin line) of the recovery of XCAT vertebrae voxel values across different noise realizations, for each individual vertebra. The XCAT activity map only has activity in the bone marrow (five times higher than the rest of the body). All noise realizations were reconstructed using 12–204 updates with 12 subsets (1i12s–17i12s).

An improvement in precision was found when including more vertebrae in the recovery calculations for the XCAT activity maps (Suppl. Fig. 3). However, the mean recovery (i.e., the accuracy of the activity quantification) decreased when including more vertebrae in the calculations. The individual recoveries for each vertebra are presented in Fig. 4, were a significant decrease (*p* < 0.05) in recovery is seen for L2–T12, over all time points p.i., compared to L3–L5. As L2–T12 are near axially aligned high-uptake regions (Fig. 5), an additional XCAT activity experiment was performed without these regions to assess their impact on vertebral recovery. In this setup, a stable mean recovery was observed across all vertebrae (Fig. 6). Calculating the ADs in the XCAT phantom using vertebrae L5–L3, results in a 58% restoration of the ground truth AD, as opposed to 21% for vertebrae L2–T12.

## Discussion

Although image-based bone marrow dosimetry is being used in an increasing number of studies, the quantitative accuracy of this method has not been fully explored. To fill this gap, we examined the relationship between the number of included vertebrae and the resulting bone marrow AD. Lower precision in bone marrow AD was observed when using fewer vertebrae, manifesting as a large AD dependency based on the cavity used in the calculation. In fact, the precision in bone marrow AD improved across all patients as the number of vertebrae increased, with the COV decreasing from 32 to 6.2% when using six instead of one vertebra. Thus, our results clearly support the inclusion of multiple lesion-free bone cavities in bone marrow AD calculations to enhance precision.

Measuring the uptake in vertebrae located near high-uptake organs proved to be challenging. A significant decrease in recovery was observed in vertebrae L1 and L2 in the XCAT phantom, which are close to the kidneys and liver. Recovery remained relatively constant when activity was placed only in the bone marrow. This suggests that closely located high-uptake organs cause underestimation of activity in adjacent low-uptake vertebrae. Similarly, only a 21% restoration of the ground truth AD dose was found in T12–L2 (biased by adjacent organs) compared to a 58% restoration in L3–L5 (significantly less bias). This highlights the direct impact of activity determination on AD calculations and emphasizes the need to consider both precision and accuracy to ensure a reliable AD assessment. In contrast, no significant differences were found when comparing the ADs between L3–L5 and T12–L2 in patients. Unlike our XCAT activity maps, metastases and high-uptake organs in patients can be more unevenly distributed due to anatomical variations, making it difficult to judge the affected vertebrae. A more caudal kidney position for example could potentially affect the lower lumbar vertebrae signal. As a result, when comparing the AD between L3–L5 and T12–L2 in patients, some vertebrae in the L3–L5 region may be affected by signal loss due to adjacent high uptake, whereas some vertebrae in the T12–L2 region may not. The natural anatomical variations in patients may partly explain why no significant difference was observed between the two groups. In the XCAT phantom, the affected vertebrae can be clearly identified; however, in patients it is difficult to determine whether differences in signal between vertebrae are caused by adjacent uptake, image noise, or physiological variations in uptake. Nevertheless, our results indicate that great care should be taken when selecting which vertebrae to include in the dose calculation, as bone marrow cavities near high-uptake regions may lead to an underestimation of the bone marrow AD.

The ADs of the patient cohort reported in the present study (mean 56.7 mGy/GBq) are consistent with previously published reports and tends to lie toward the upper end of the range [2, 4, 5, 7, 17, 22]. In these prior studies, no substantial differences in mean AD have been reported between studies using one versus multiple vertebrae in their AD calculations. This is in good agreement with our findings, where no major differences in mean AD across patients were observed between analyses based on one and six vertebrae (Table 2). However, as convincingly supported by the present data, the precision of the AD improved considerably when multiple vertebrae were used.

Partial volume corrections are commonly applied to organs-at-risk in radionuclide therapy, but not to the bone marrow (Table 1). One single study did use partial volume correction for the bone marrow, by determining the RC in an 80 mL insert filled with 0.03 MBq/mL [15]. The RC was then applied to the activity estimations from the L2–L4 vertebrae. However, applying a constant partial volume correction is challenging at these high noise levels. Our results show that the RC varies considerably depending on volume and noise level. When delineating smaller volumes, such as the L2–L4 vertebrae, the RC shows significant variation depending on the specific noise realization being analyzed. Other studies have used a small VOI placed within the vertebrae [7, 23], stating that partial volume effects are negligible due to the homogeneous distribution in the vertebrae surrounding the VOI. However, since noise levels and spill-over effects within the vertebrae may disrupt this homogeneity, it is reasonable to assume that a smaller VOI may lead to a higher noise-dependency–and thus lower precision. Further studies are needed to clarify how the selected VOI size in image-based bone marrow dosimetry affects the derived activity estimates. A recovery < 1 in all our activity quantification experiments suggests that applying partial volume correction could help mitigate spill-out effects caused by the limited resolution of SPECT, potentially improving accuracy. The potential for underestimating bone marrow ADs when vertebrae are adjacent to high-uptake regions, combined with the risk of overestimation due to bone metastases, as well as the noise challenges discussed above, makes the application of a constant partial volume correction to the bone marrow particularly challenging. Further work is needed to correctly compensate for the loss of signal near high-uptake structures.

Due to the limited spatial resolution of low-dose CT, delineation of the vertebral bone marrow can be challenging. Although the vertebral body typically has a relatively large volume [24], the marrow volume that can be reliably delineated on low-dose CT is limited (median VOI size ranging from 4.6–10.1 mL (T10–L5); Supplementary Data Fig. 4). Other source regions have been used for image-based bone marrow dosimetry, such as the pelvic bone and humerus [2, 25]. A potential advantage of these regions compared with vertebral cavities may be a reduced contribution of signal loss from adjacent high-uptake organs, given their anatomical positioning. However, in clinical practice, the limited FOV of SPECT acquisitions may restrict or even preclude delineation of these skeletal regions.

The observed signal loss in vertebrae adjacent to high-uptake regions likely reflects a reconstruction-related bias, where low-uptake regions cannot be adequately resolved when located near areas of high uptake. Comparing the recovery curves in the XCAT phantoms of L5 and L1, L5 appears to reach convergence while L1 gradually increases across the 204 updates, suggesting that the behavior is reconstruction-dependent. Although the signal loss in vertebrae adjacent to high-uptake regions is more evident at a lower number of updates, the progressive reduction in precision with increasing updates indicates that a restrained number of updates is more suitable for image-based bone marrow dosimetry. Therefore, consistent with previous bone marrow dosimetry studies, 60 updates were used in this study. The number of subsets is often between 8 and 10, and reconstructing high-noise projections with fewer subsets is preferred [14]. We examined reducing the number of subsets from 12 to 1, but no significant effects on bone marrow AD were observed. Similarly, only small differences were seen when comparing 1 subset (204i1s) and 12 subsets (17i12s), for the Lung-Spine phantom at the highest noise levels examined. However, it should be noted that these findings were obtained using our in-house Monte Carlo code, and further validation is required for other reconstruction algorithms.

### Limitations

One limitation of our study is that one and the same volume fraction, representing the proportion of active red marrow in the vertebrae, was assumed for all patients in AD calculations. In reality, both the composition and structure of bone marrow are known to vary with age, where the fraction of active red marrow decreases with increasing age [26]. As the patient cohort in this study spans a relatively wide age range, the true volume fraction is therefore likely to vary between individuals, resulting in inaccuracies in our AD estimates. To accurately estimate the AD to the bone marrow, patient-specific bone marrow distributions are needed. Methods for localizing active bone marrow in patients have been proposed [27, 28], and incorporating such approaches in future studies could improve the accuracy of AD estimates.

In the present study, the absorbed dose calculations were restricted to the electron component from ^177^Lu. This simplification was made to isolate the impact of vertebral sampling on the precision of the absorbed dose estimates. Although photon cross dose from nearby organs or lesions will contribute to the total bone marrow dose [6, 29], this component is generally more spatially distributed and was therefore not assumed to influence the relative comparisons between different vertebral combinations investigated in this work.

Another limitation is the assumption of homogeneous activity distributions within organs and background compartments in the XCAT phantom. Also, no lesions were added to the XCAT phantom. In patients, the radiotracer uptake is typically heterogeneous, both within organs and in surrounding tissues, and lesions are often present within the SPECT FOV. The activity distribution of the XCAT phantoms was designed to represent a typical patient, achieved by selecting a patient whose organ activity concentrations closely matched the cohort median. As the objective was to model a representative rather than patient-specific activity distributions, lesions were not included in the activity maps. However, as our findings clearly demonstrate the influence of high-uptake regions on activity estimates in vertebral bone marrow, future studies should investigate the impact of lesion size and spatial positioning on bone marrow activity estimation.

## Conclusion

We highlight for the first time the importance of including multiple lesion-free vertebrae when conducting image-based bone marrow dosimetry to enhance precision. However, careful selection of vertebrae is crucial, as adjacent high-uptake regions and partial volume effects can bias the absorbed dose estimation. This suggests that a trade-off must be found between including as many vertebrae as possible (to increase precision) while excluding vertebrae that are strongly biased by adjacent high-uptake regions (to improve accuracy). Furthermore, to improve accuracy, our findings suggest that applying partial volume correction could help mitigate signal loss in vertebrae. Finally, reducing the number of subsets used in the reconstruction from 12 to 1 did not significantly impact bone marrow absorbed dose estimation.

## Supplementary Information

Additional file1 (DOCX 5725 kb)

## Data Availability

The data used and analyzed in the study is available from the corresponding author on reasonable request.
