# Supplementary material for: Multiple vertebrae improves precision in image-based bone marrow absorbed dose estimation in [177Lu]Lu–DOTATATE treatment
Source: EJNMMI Phys. 2026 May 9;13:43. doi: 10.1186/s40658-026-00882-4 (PMC13161456; doi:10.1186/s40658-026-00882-4)
Supplement: Supplementary file 1 — Additional file1 (DOCX 5725 kb) [file 40658_2026_882_MOESM1_ESM.docx]

**Supplementary Data**

**FIGURE 1** Illustrative plot of the generated unique combinations for the bone marrow absorbed dose calculations in patients with 10 delineated bone cavities.

**FIGURE 2** Mean recovery coefficient (thick line) and standard deviation (thin line) of the recovery coefficient for noise level η_max_ in the Lung-Spine phantom, over the three different sphere volumes. The blue lines show the RC when using one subset (60i1s), and the red lines show the RC when using 12 subsets (5i12s), both at 60 updates.


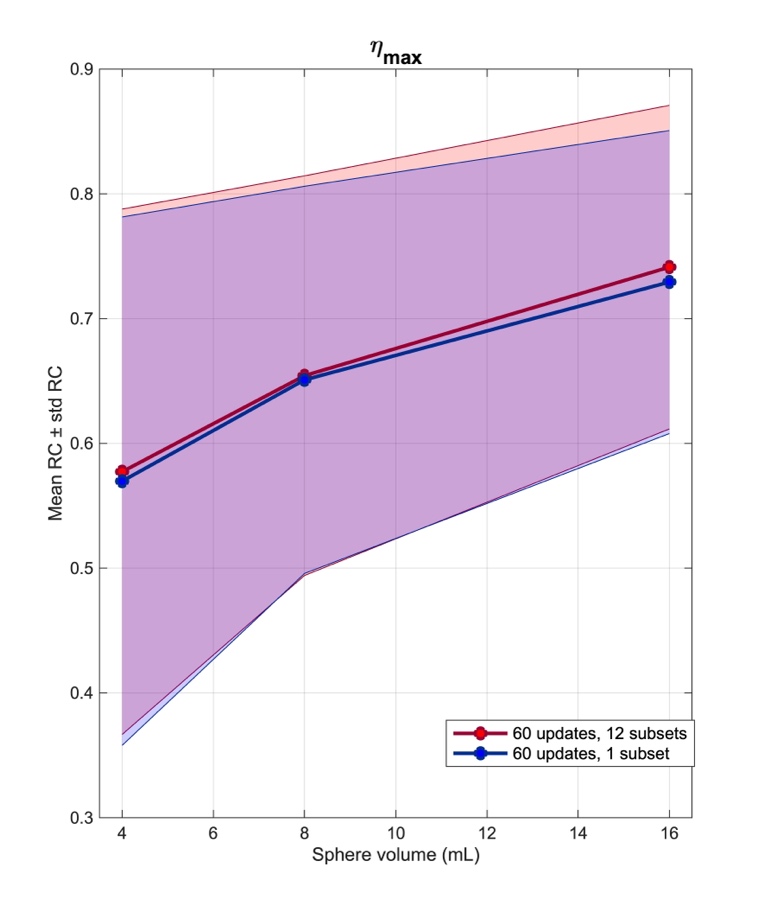


**FIGURE 3** Mean recovery (thick line) and standard deviation (thin line) of the recovery of XCAT vertebrae voxel values across different noise realizations, with an increasing number of included vertebrae used in the recovery calculations (left to right). Four XCAT activity maps **A)** 2 h p.i., **B)** 24 h p.i., **C)** 48 h p.i., and **D)** 168 h p.i. are presented. All noise realizations were reconstructed using 12 to 204 updates, with 12 subsets (1i12s-17i12s, respectively).


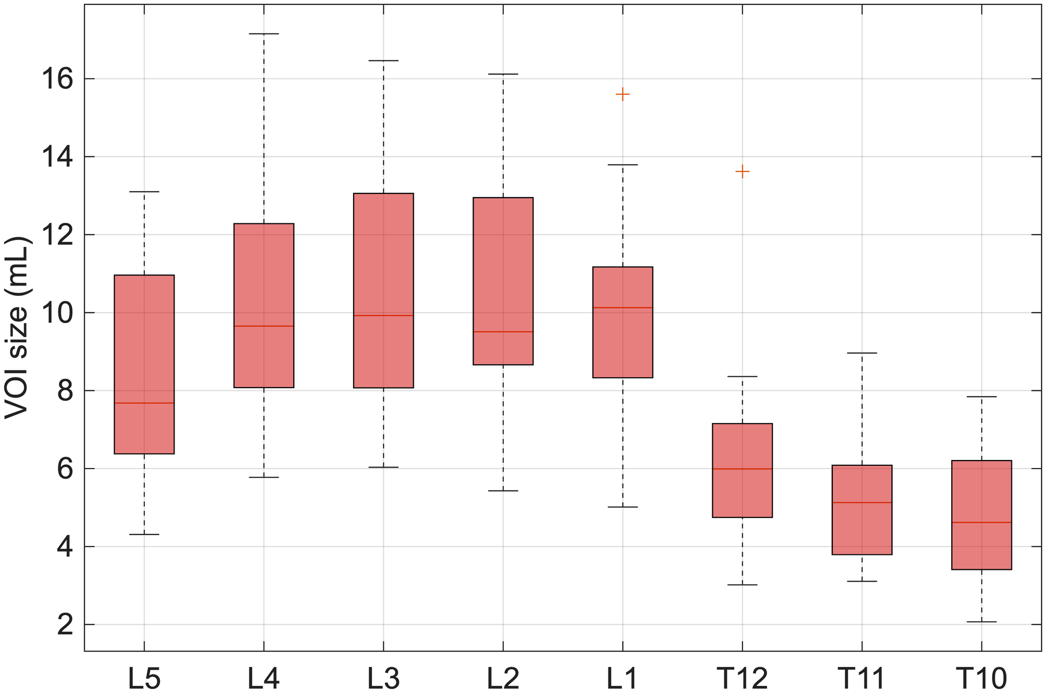


**FIGURE 4** A boxplot showing the variation in VOI size (mL) of vertebrae T10-L5, delineated on the CT acquired 2 hours post injection.

**Tables**

**TABLE 1** The activity concentration (C) and time per projections for the three different noise levels.

|  | **C(MBq/mL)** | **Time per projection (s)** |
| --- | --- | --- |
| **η_min_** | 0.045 | 2.28 |
| **η_med_** | 0.020 | 1.02 |
| **η_max_** | 0.005 | 0.28 |

**TABLE 2** Four activity maps (2, 24, 48, 168 h p.i.) modelled into the XCAT phantom. The table shows the relative mean voxel values used.

| **Time points**  **(h p.i.)** | **Liver** | **Left Kidney** | **Right Kidney** | **Spleen** | **Bone Marrow** | **Lungs** | **Background** |
| --- | --- | --- | --- | --- | --- | --- | --- |
| 2 | 134 | 845 | 827 | 557 | 23 | 19 | 9 |
| 24 | 95 | 771 | 756 | 594 | 21 | 7 | 4 |
| 48 | 68 | 490 | 494 | 409 | 20 | 7 | 3 |
| 168 | 19 | 73 | 70 | 126 | 5 | 2 | 1 |

**TABLE 3** Mean bone marrow absorbed doses for each patient, calculated using one to six vertebrae with 60 updates, 12 subsets (5i12s), are presented in mGy/GBq. The precision, expressed as the coefficient of variation (COV), is provided in parentheses. The variable n denoted the number of vertebrae delineated on each patient.

| **Dose (COV) [mGy]** | | | | | | | |
| --- | --- | --- | --- | --- | --- | --- | --- |
|  | **One Vert.** | **Two Vert.** | **Three Vert.** | **Four Vert.** | **Five Vert.** | **Six Vert.** |  |
| **Pat A (n=8)** | 55.9 (14%) | 54.9 (9%) | 54.5 (6.8%) | 54.4 (5.3%) | 54.3 (4.2%) | 54.2 (3.0%) |  |
| **Pat B (n=8)** | 38.6 (40%) | 36.7 (24%) | 36.4 (17%) | 36.2 (12%) | 36.1 (9.5%) | 36.2 (6.9%) |  |
| **Pat C (n=7)** | 90.0 (29%) | 92.8 (21%) | 91.7 (14%) | 91.5 (10%) | 91.2 (7.4%) | 90.9 (5.0%) |  |
| **Pat D (n=9)** | 62.7 (22%) | 62.8 (16%) | 62.6 (12%) | 62.4 (9.4%) | 62.3 (7.6%) | 62.2 (6.0%) |  |
| **Pat E (n=9)** | 72.5 (35%) | 70.1 (23%) | 69.6 (17%) | 69.5 (13%) | 69.2 (11%) | 69.2 (8.4%) |  |
| **Pat F (=7)** | 62.8 (18%) | 62.9 (12%) | 62.7 (8.7%) | 62.7 (6.6%) | 62.8 (4.6%) | 63.0 (2.8%) |  |
| **Pat G (n=7)** | 82.7 (33%) | 81.0 (20%) | 79.3 (15%) | 79.4 (11%) | 79.5 (8.2%) | 79 (5.5%) |  |
| **Pat H (n=9)** | 70.2 (52%) | 54.6 (16%) | 54.9 (15%) | 54.1 (9.2%) | 54.0 (7.3%) | 53.9 (5.8%) |  |
| **Pat I (n=8)** | 44.5 (30%) | 43.8 (18%) | 43.8 (13%) | 43.8 (10%) | 43.8 (8.0%) | 43.8 (5.5%) |  |
| **Pat J (n=9)** | 41.2 (24%) | 39.8 (15%) | 39.3 (11%) | 39.2 (9.0%) | 39.2 (7.1%) | 39.1 (5.7%) |  |
| **Pat K (n=10)** | 47.1 (40%) | 44.6 (26%) | 44.3 (20%) | 44.2 (16%) | 44.1 (13%) | 44.1 (11%) |  |
| **Pat L (n=10)** | 44.9 (46%) | 40.4 (14%) | 39.7 (10%) | 39.4 (8.5%) | 39.3 (7.0%) | 39.3 (5.7%) |  |
| **Pat M (n=10)** | 39.9 (24%) | 38.2 (14%) | 37.8 (11%) | 37.7 (8.7%) | 37.6 (7.0%) | 37.5 (5.7%) |  |
| **Pat N (n=10)** | 42.8 (34%) | 41.6 (20%) | 41.6 (15%) | 41.5 (12%) | 41.5 (10%) | 41.4 (8.0%) |  |
| **Pat O (n=9)** | 50.5 (35%) | 49.0 (21%) | 48.9 (15%) | 48.8 (12%) | 48.6 (10%) | 48.6 (7.5%) |  |
| **Pat P (n=8)** | 105 (43%) | 102 (25%) | 101 (18%) | 101 (14%) | 101 (11%) | 101 (7.6%) |  |
| **Mean** | *59.5 (32%)* | *57.2 (18%)* | *56.8 (14%)* | *56.6 (11%)* | *56.5 (8.2%)* | *56.5 (6.3%)* |  |

**TABLE 4** The ratio of the mean absorbed doses for each patient, calculated as the absorbed doses calculated using 12 subsets (5i12s) divided by the absorbed doses calculated using one subset (60i1s), for each included vertebra.

| **Absorbed Dose Ratio Table** (60 updates, 12 subsets divided by one subset) | | | | | | |
| --- | --- | --- | --- | --- | --- | --- |
|  | **One Vert.** | **Two Vert.** | **Three Vert.** | **Four Vert.** | **Five Vert.** | **Six Vert.** |
| **Pat A** | 100% | 99% | 99% | 99% | 99% | 99% |
| **Pat B** | 103% | 104% | 104% | 104% | 104% | 103% |
| **Pat C** | 100% | 100% | 100% | 100% | 100% | 100% |
| **Pat D** | 97% | 98% | 98% | 98% | 97% | 97% |
| **Pat E** | 98% | 97% | 97% | 97% | 97% | 97% |
| **Pat F** | 99% | 99% | 99% | 99% | 99% | 99% |
| **Pat G** | 97% | 97% | 97% | 97% | 97% | 97% |
| **Pat H** | 100% | 99% | 101% | 100% | 100% | 100% |
| **Pat I** | 98% | 98% | 98% | 98% | 98% | 98% |
| **Pat J** | 106% | 104% | 103% | 103% | 103% | 103% |
| **Pat K** | 101% | 101% | 101% | 101% | 101% | 101% |
| **Pat L** | 103% | 99% | 100% | 100% | 100% | 100% |
| **Pat M** | 101% | 99% | 99% | 99% | 99% | 99% |
| **Pat N** | 101% | 100% | 100% | 100% | 100% | 100% |
| **Pat O** | 101% | 101% | 100% | 101% | 100% | 100% |
| **Pat P** | 101% | 101% | 101% | 101% | 101% | 101% |

**TABLE 5** Mean recovery coefficient and coefficient of variation (COV) (presented in parentheses) of the noise levels η_min_- η_max_, for the three different sphere sizes (4–16 mL), in the Lung-Spine phantom. The noise realizations are all reconstructed using 12-204 updates, with 12 subsets (1i12s-17i12s).

|  |  | **12** | **24** | **36** | **48** | **60** | **72** | **84** | **96** |  |
| --- | --- | --- | --- | --- | --- | --- | --- | --- | --- | --- |
| **η_min_** | | **16 mL** | 0.61 (3%) | 0.66 (4%) | 0.71 (5%) | 0.73 (5%) | 0.74 (5%) | 0.75 (5%) | 0.76 (6%) | 0.76 (6%) |
|  |  | **8 mL** | 0.56 (4%) | 0.60 (6%) | 0.64 (7%) | 0.66 (8%) | 0.68 (8%) | 0.69 (9%) | 0.70 (9%) | 0.70 (9%) |
|  |  | **4 mL** | 0.44 (5%) | 0.51 (8%) | 0.56 (10%) | 0.59 (11%) | 0.62 (12%) | 0.64 (13%) | 0.66 (14%) | 0.67 (15%) |
|  | |  |  |  |  |  |  |  |  |  |
| **η_med_** | | **16 mL** | 0.61 (5%) | 0.66 (7%) | 0.70 (8%) | 0.72 (8%) | 0.74 (9%) | 0.74 (9%) | 0.75 (9%) | 0.75 (9%) |
|  |  | **8 mL** | 0.57 (6%) | 0.61 (9%) | 0.64 (11%) | 0.67 (13%) | 0.68 (14%) | 0.69 (15%) | 0.70 (16%) | 0.70 (16%) |
|  |  | **4 mL** | 0.45 (8%) | 0.51 (13%) | 0.55 (16%) | 0.59 (19%) | 0.61 (21%) | 0.64 (22%) | 0.66 (23%) | 0.67 (24%) |
|  | |  |  |  |  |  |  |  |  |  |
| **η_max_** | | **16 mL** | 0.63 (9%) | 0.68 (13%) | 0.71 (17%) | 0.73 (17%) | 0.74 (17%) | 0.75 (18%) | 0.75 (18%) | 0.75 (19%) |
|  |  | **8 mL** | 0.57 (11%) | 0.60 (16%) | 0.63 (22%) | 0.64 (22%) | 0.65 (24%) | 0.66 (26%) | 0.67 (27%) | 0.67 (28%) |
|  |  | **4 mL** | 0.46 (13%) | 0.50 (22%) | 0.53 (28%) | 0.56 (33%) | 0.58 (36%) | 0.59 (39%) | 0.60 (42%) | 0.61 (44%) |
|  | |  |  |  |  |  |  |  |  |  |
|  | |  |  |  |  |  |  |  |  |  |
|  | | **108** | **120** | **132** | **144** | **156** | **168** | **180** | **192** | **204** |
| **η_min_** | | 0.76 (6%) | 0.76 (6%) | 0.76 (6%) | 0.77 (6%) | 0.77 (6%) | 0.77 (6%) | 0.77 (6%) | 0.77 (6%) | 0.77 (6%) |
|  |  | 0.71 (9%) | 0.71 (10%) | 0.71 (10%) | 0.72 (10%) | 0.72 (10%) | 0.72 (10%) | 0.72 (10%) | 0.72 (10%) | 0.72 (10%) |
|  |  | 0.68 (16%) | 0.69 (16%) | 0.69 (16%) | 0.70 (17%) | 0.70 (17%) | 0.70 (18%) | 0.70 (18%) | 0.70 (18%) | 0.70 (18%) |
|  | |  |  |  |  |  |  |  |  |  |
| **η_med_** | | 0.75 (10%) | 0.75 (10%) | 0.75 (10%) | 0.75 (10%) | 0.75 (10%) | 0.75 (10%) | 0.75 (10%) | 0.75 (10%) | 0.75 (10%) |
|  |  | 0.71 (17%) | 0.71 (17%) | 0.71 (18%) | 0.71 (18%) | 0.72 (18%) | 0.72 (18%) | 0.72 (19%) | 0.72 (19%) | 0.72 (19%) |
|  |  | 0.68 (25%) | 0.69 (25%) | 0.69 (26%) | 0.70 (26%) | 0.70 (27%) | 0.71 (27%) | 0.71 (28%) | 0.71 (29%) | 0.71 (29%) |
|  | |  |  |  |  |  |  |  |  |  |
| **η_max_** | | 0.75 (19%) | 0.75 (19%) | 0.75 (19%) | 0.75 (19%) | 0.75 (19%) | 0.75 (19%) | 0.75 (20%) | 0.75 (20%) | 0.75 (20%) |
|  |  | 0.67 (29%) | 0.67 (29%) | 0.67 (30%) | 0.68 (30%) | 0.68 (31%) | 0.68 (31%) | 0.68 (31%) | 0.68 (32%) | 0.68 (32%) |
|  |  | 0.61 (46%) | 0.61 (47%) | 0.61 (49%) | 0.62 (50%) | 0.62 (51%) | 0.62 (52%) | 0.62 (54%) | 0.62 (54%) | 0.62 (55%) |

**TABLE 6** Mean recovery coefficient and coefficient of variation (COV) (presented in parentheses) of noise level η_max_, for the three different sphere sizes (4–16 mL), in the Lung-Spine phantom. The noise realizations are all reconstructed using 12-204 updates, with 1 subset (12i1s-204i1s).

|  |  | **12** | **24** | **36** | **48** | **60** | **72** | **84** | **96** |  |
| --- | --- | --- | --- | --- | --- | --- | --- | --- | --- | --- |
| **η_max_** | | **16 mL** | 0.62 (8%) | 0.67 (13%) | 0.70 (15%) | 0.72 (16%) | 0.73 (17%) | 0.73 (17%) | 0.74 (17%) | 0.74 (18%) |
|  | **8 mL** | 0.57 (10%) | 0.59 (16%) | 0.62 (19%) | 0.64 (22%) | 0.65 (24%) | 0.65 (25%) | 0.66 (26%) | 0.67 (27%) |  |
|  | **4 mL** | 0.45 (13%) | 0.49 (22%) | 0.53 (29%) | 0.55 (33%) | 0.57 (37%) | 0.58 (40%) | 0.59 (42%) | 0.60 (45%) |  |
|  |  |  |  |  |  |  |  |  |  |  |
|  |  |  |  |  |  |  |  |  |  |  |
|  | **108** | **120** | **132** | **144** | **156** | **168** | **180** | **192** | **204** |  |
|  | 0.74 (18%) | 0.74 (18%) | 0.74 (18%) | 0.74 (18%) | 0.74 (19%) | 0.74 (19%) | 0.74 (19%) | 0.74 (19%) | 0.74 (19%) |  |
|  | 0.67 (29%) | 0.67 (29%) | 0.67 (29%) | 0.67 (29%) | 0.67 (30%) | 0.68 (30%) | 0.68 (30%) | 0.68 (31%) | 0.68 (31%) |  |
|  | 0.60 (46%) | 0.61 (48%) | 0.61 (49%) | 0.61 (51%) | 0.61 (52%) | 0.61 (53%) | 0.61 (54%) | 0.61 (55%) | 0.61 (55%) |  |
